# The Efficacy and Health Economics of Different Treatments for Type 1 Cesarean Scar Pregnancy

**DOI:** 10.3389/fphar.2022.822319

**Published:** 2022-01-28

**Authors:** Tingting Hong, Zeying Chai, Manman Liu, Lingzhi Zheng, Feng Qi

**Affiliations:** Department of Gynecology and Obstetrics, Taizhou Hospital of Zhejiang Province Affiliated to Wenzhou Medical University, Linhai, China

**Keywords:** cesarean scar pregnancy, ultrasound-guided, methotrexate injection, lauromacrogol injection, suction aspiration, uterine artery embolization

## Abstract

**Objectives:** To evaluate the efficacy and health economics of four treatments for type 1 cesarean scar pregnancy (CSP).

**Methods:** From January 2009 to December 2018, 326 patients diagnosed with type 1 CSP were examined, among whom 31 received ultrasound-guided local injection of methotrexate (local injection group), 160 patients received uterine artery embolization combined with suction aspiration (UAE group), 25 patients received ultrasound-guided suction aspiration (aspiration group) and 90 received ultrasound-guided local injection of lauromacrogol combined with suction aspiration (lauromacrogol group). Clinical data and outcomes were analyzed. The decision tree model was used to compare the economics of four treatments.

**Results:** The success rate of the local injection group was 71.0% (22/31), which was significantly different from 98.8% (158/160) of the UAE group and 100.0% (90/90) of the lauromacrogol group. The success rate of the aspiration group was 92.0% (23/25), which was significantly lower than that of the lauromacrogol group. The cost-effectiveness ratio was 1,876.53 yuan for the aspiration group, 2,164.63 yuan for the lauromacrogol group, 4,383.56 yuan for the local injection group, and 7,850.81 yuan for the UAE group. The Incremental cost effectiveness ratio (ICER) of the lauromacrogol group to the aspiration group was 5,477.75 yuan, indicating that if the willing to pay of patients was higher than 5,477.75 yuan, the lauromacrogol group had a cost-effectiveness advantage in treating type 1 CSP, compared to aspiration group. On the contrary, aspiration group has a higher cost-effectiveness advantage. The ICER of the lauromacrogol group to the local injection group or the UAE group were both less than 0, indicating that local injection group and UAE group was not cost-effective in the treatment of type 1 CSP.

**Conclusion:** For type 1 CSP, the ultrasound-guided local injection of lauromacrogol combined with suction aspiration and ultrasound-guided suction aspiration, are effective and economical, and the choice between the two can be based on the patient’s willing to pay.

## 1 Introduction

Cesarean scar pregnancy (CSP) refers to the implantation of the gestational sac within the scar of the previous cesarean surgery and is one of the long-term complications of a cesarean section (c-section). If not detected early and treated in time, it can lead to placenta accreta spectrum (PAS), massive vaginal bleeding, hysterectomy, and even maternal death (Ash et al., 2007; Miller et al., 2020). The incidence of CSP is approximately between 1:1800 and 1:2,216 (Jurkovic et al., 2003; Seow et al., 2004; Timor-Tritsch et al., 2019b). Due to the high rate of c-section deliveries in the previous decades, wide use of transvaginal ultrasound, and abandoning of the family planning policy in China, the number of patients with CSP has increased rapidly in recent years (Lumbiganon et al., 2010; Birch Petersen et al., 2016).

The current treatment methods for CSP include local or systemic administration of methotrexate, ultrosound-guided aspiration, hysteroscopic resection, laparoscopic, transvaginal or transabdominal resection or hysterectomy, and adjuvant treatments such as local injection of lauromacrol, uterine artery embolization, balloon compression, and high-intensity focused ultrasound, which can be combined with drug therapy or surgical treatment to manage CSP (Birch Petersen et al., 2016; Maheux-Lacroix et al., 2017; Chai et al., 2018; Timor-Tritsch et al., 2019a). However, there is no optimal treatment strategy yet.

There are two types of CSP based on the classification by Vial et al. (Vial et al., 2000): type 1 is the chorionic villi that is implanted on the scar and grows toward the cervical or uterine cavity, and type 2 is a deep implantation of gestational tissue in the scar that grows toward the bladder. As different type of CSP will affect the choice of treatment, here we sampled patients with type 1 CSP who received treatment in Taizhou Hospital in Zhejiang Province of China from January 2009 to December 2018, and evaluated the efficacy and health economics of treatments.

## 2 Materials and Methods

### 2.1 Patients

This study was approved by the ethics committee of Taizhou Hospital of Zhejiang Province affiliated to Wenzhou Medical University.

The criteria for including patients in the study were as follows: 1) history of previous cesarean delivery; 2) positive pregnancy test; 3) transvaginal ultrasound (TVUS) imaging indicating CSP according to the diagnostic criteria recommended by Timor-Tritsch et al. (Miller et al., 2020); 4) type 1 CSP; 5) absence of any active inflammation; 6) patient not having received treatment for any other disorder unrelated to CSP during hospitalization; and 7) availability of complete clinical data. Before starting the treatment, each patient was informed of the effects and potential risks of different treatments and was asked to select a treatment plan and provide written consent.

A total of 326 patients with type 1 CSP were enrolled, among whom 31 received ultrasound-guided local injection of methotrexate (MTX); 160 received uterine artery embolization combined with suction aspiration; 25 received ultrasound-guided suction aspiration; 90 received ultrasound-guided local injection of lauromacrogol combined with suction aspiration; seven received lapalotomy and resection; three received systemic administration of MTX; two received hysteroscopy-assisted laparoscopy; and eight received hysteroscopy. Treatments of fewer than 10 patients were eliminated to reduce sample bias. The efficacy and health economics of the four treatments, including ultrasound-guided local injection of MTX (local injection group), uterine artery embolization combined with suction aspiration (UAE group), ultrasound-guided suction aspiration (aspiration group), and ultrasound-guided local injection of lauromacrogol combined with suction aspiration (lauromacrogol group), were compared.

### 2.2 Procedures

#### 2.2.1 Ultrasound-Guided Local Injection of Lauromacrogol Combined With Suction Aspiration

Detailed technical steps of this treatment method have been described in a previous publication (Chai et al., 2018). In this study, aspiration was performed under the guidance of abdominal ultrasound, 12–24 h after multi-point injecting of lauromacrogol around the peritrophoblastic tissue.

#### 2.2.2 Ultrasound-Guided Suction Aspiration

The cervix was dilated to 7.5 cm using Hegar dilators, and the gestational mass was removed via suction aspiration, under the guidance of abdominal ultrasound.

#### 2.2.3 Uterine Artery Embolization Combined With Suction Aspiration

The detailed procedure of UAE has been reported previously (Qi et al., 2015). The uterine arteries were embolized with 1–2 mm gelfoam particles bilaterally, and ultrasound-guided suction aspiration was performed 24–120 h later.

#### 2.2.4 Ultrasound-Guided Local Injection of MTX

Under ultrasound guidance a 21-gauge needle (Hakko, Tokyo, Japan) was inserted into the gestational sac and a 50 mg dose of MTX was slowly injected after the fluid in the gestational sac was aspirated.

### 2.3 Health Economic Method

**FIGURE 1:**
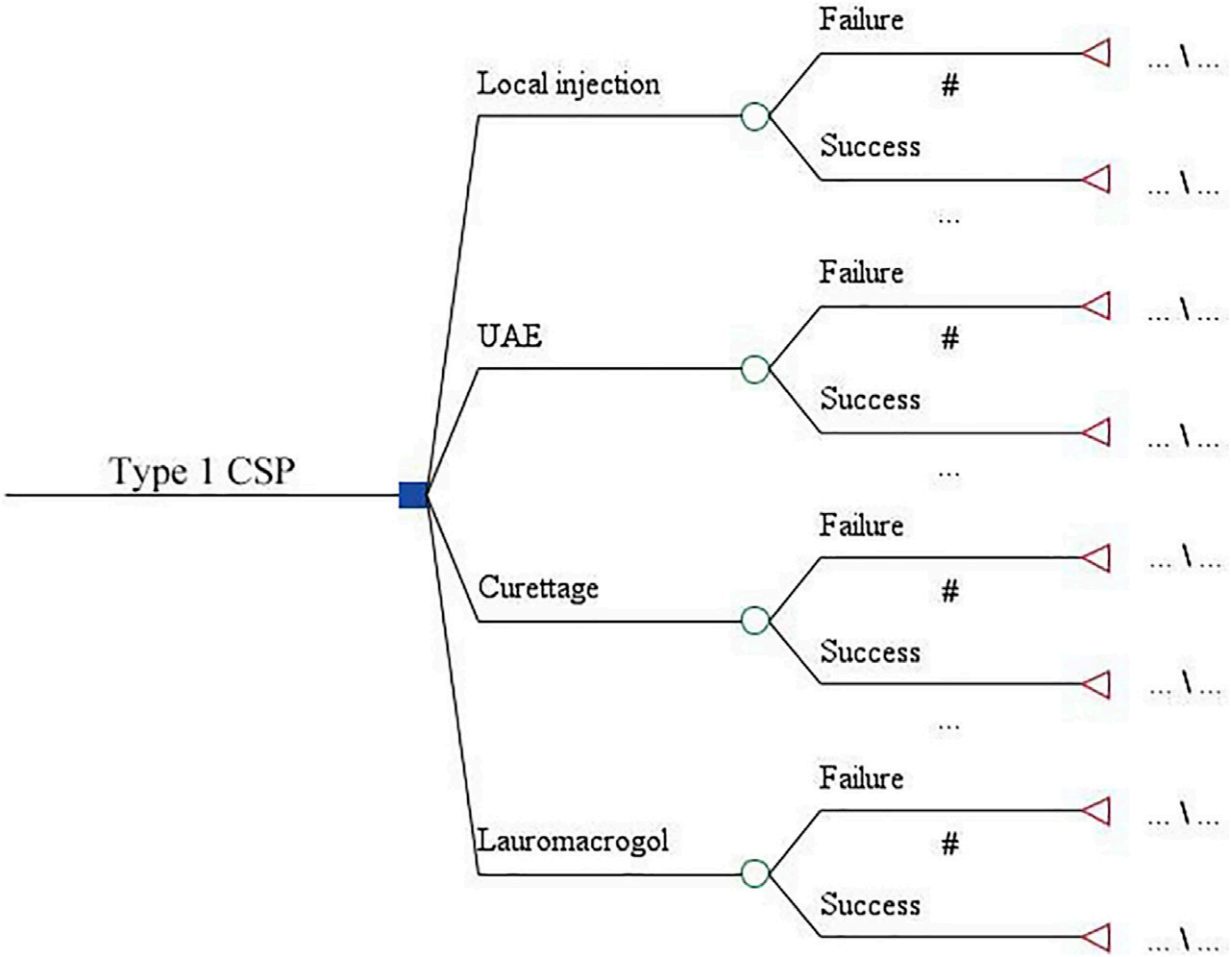
Decision tree model to evaluate the economics of four treatments for type 1 CSP.

The decision tree model was used to compare the economics of four treatments for type 1 CSP. The model consists of four branches representing the local injection, UAE, aspiration, and lauromacrogol groups. Two sub-branches were derived from each regimen, representing the success or failure of each treatment. The time span of the model was from diagnosis to cure (Figure 1).

### 2.4 Model Parameters

1 Total treatment cost = direct medical cost + direct non-medical cost + indirect cost + hidden cost. Direct non-medical costs are subjective and vary greatly, and indirect and hidden costs are difficult to calculate and greatly influence the results. Therefore, only direct medical costs are considered, including medical service fees, examination fees (laboratory, imaging), treatment fees, drug fees and so on.
2 The value of effect was the treatment success rate of CSP. Criteria: Weekly ultrasound examination showed no remnant and serum β-human chorionic gonadotropin (β -HCG) decreased to normal (<5 IU/L). The recovery time of menstruation was recorded. Vaginal bleeding >200 ml during treatment or β -HCG decrease ≤50% by the seventh day post-treatment were considered as treatment failure. The salvage therapy included Foley catheter balloon compression, ultrasound-guided suction aspiration, or uterine artery embolization.
3 This study was based on the decision tree model to conduct cost-effectiveness analysis of each treatment for CSP. Incremental cost effectiveness ratio (ICER) was used as the criteria. The ICER and willing to pay (WTP) value are compared to judge the economy of therapies. When there was no statistical difference in the effects, the special method of cost-effectiveness analysis (minimum cost analysis) was used for the evaluation of the economy of treatments.
4 The robustness of the results was evaluated by univariate sensitivity and probabilistic sensitivity analyses. The univariate sensitivity analysis was represented by the tornado diagram, and the probabilistic sensitivity analysis was represented by the cost-effectiveness acceptable curve. It is assumed that the fluctuation range of cost is a 95% confidence interval (CI), and the fluctuation range of effect is 10%. If the fluctuation range exceeds 100%, it will be calculated as 100%.

### 2.5 Data Analysis

The data analyses were performed by SPSS 23.0 (IBM Inc. Armonk, NY). Continuous data were described by mean ± standard deviation (±S), and categorical data were described by percentage. All *p* values are bilateral probability, and the difference was statistically significant at *p* values <0.05. For data conforming to the normal distribution, the analysis of variance (ANOVA) was used to compare continuous data. The χ^2^ test or Fisher’s exact test was used to compare the categorical data. The non-parametric test (Mann-Whitney *U* test) was used for data not conforming to the normal distribution. Treeage Pro 2011 (TreeAge Pro Inc., Williamston, MA) was used to build the decision tree model, set the model parameters, calculate ICER, and conduct the sensitivity analysis by setting the variation range of each parameter.

## 3 Results

**FIGURE 2:**
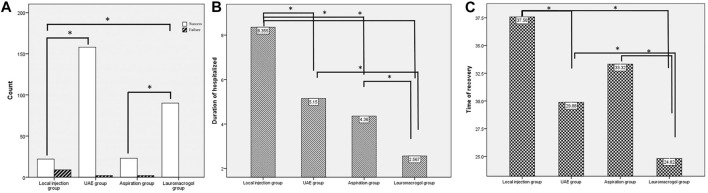
**(A)** The success rate of the local injection group was 71.0% (22/31), which was significantly different from 98.8% (158/160) of the UAE group and 100.0% (90/90) of the lauromacrogol group. The success rate of the aspiration group was 92.0% (23/25), which was significantly lower than that of the lauromacrogol group. **(B)** The length of hospital stay in the lauromacrogol group was the shortest (2.57 ± 1.01 days), which was significantly different from those of the local injection group (8.35 ± 4.36 days), UAE group (5.15 ± 2.07 days), and aspiration group (4.36 ± 2.38 days). The length of hospital stay in the local injection group was significantly longer than UAE group and aspiration group. **(C)** The recovery time in the lauromacrogol group was the shortest (24.82 ± 8.51 days), which was significantly different from that of the local injection group (37.58 ± 21.48 days), UAE group (29.88 ± 10.02 days), and aspiration group (33.32 ± 13.34 days).

There were no significant differences in age, number of cesarean sections, time since previous c-section, fetal heartbeat ratio, and myometrium thickness, but significant differences were observed in serum β -HCG, gestational age, and pregnancy sac diameter. The success rate of the local injection group was 71.0% (22/31), which was significantly different from 98.8% (158/160) of the UAE group and 100.0% (90/90) of the lauromacrogol group. The success rate of the aspiration group was 92.0% (23/25), which was significantly lower than that of the lauromacrogol group. The length of hospital stay in the lauromacrogol group was the shortest (2.57 ± 1.01 days), which was significantly different from those of the local injection group (8.35 ± 4.36 days), UAE group (5.15 ± 2.07 days), and aspiration group (4.36 ± 2.38 days). The length of hospital stay in the local injection group was significantly longer than UAE group and aspiration group. The recovery time in the lauromacrogol group was the shortest (24.82 ± 8.51 days), which was significantly different from that of the local injection group (37.58 ± 21.48 days), UAE group (29.88 ± 10.02 days), and aspiration group (33.32 ± 13.34 days). There was no statistical significance in blood loss and menstrual recovery among the four groups (Table 1 and Figure 2).

**TABLE 1:** Comparison of therapeutic effects of 4 treatments for type 1 cesarean scar pregnancy.

		Local injection group	UAE group	Aspiration group	Lauromacrogol group	P
No. of cases		31	160	25	90	
Age (years)		32.7 ± 4.8	33.1 ± 5.1	32.4 ± 5.5	34.4 ± 5.0	0.125
No. of cesarean (%)	1	61.3 (19)	60.0 (96)	72 (18)	46.7 (42)	0.071
	>1	38.7 (12)	40.0 (64)	28 (7)	53.3 (48)	
Time from previous CS		6.1 ± 4.3	5.7 ± 3.8	6.4 ± 4.5	6.0 ± 3.4	0.784
β-hCG (IU/L)		16,698.5 ± 15,389.7	37,474.4 ± 39,464.9	25,374.8 ± 26,121.2	40,949.3 ± 44,074.2	0.010
Gestational age (days)		46.1 ± 8.3	51.1 ± 12.3	49.6 ± 10.6	46.2 ± 7.1	0.002
Diameter of the sac (mm)		16.7 ± 8.9	26.0 ± 14.8	21.2 ± 10.2	22.0 ± 8.7	0.001
Fetal heart activity (%)		19.4 (6)	39.2 (60)	28.0 (7)	42.2 (38)	0.095
Thickness of myometrium (mm)		4.0 ± 1.5	4.1 ± 1.3	4.6 ± 1.8	4.3 ± 1.0	0.321
Success rate (%)		71.0 (22)	98.8 (158)	92.0 (23)	100.0 (90)	<0.001
Time of recovery (days)		37.6 ± 21.5	29.9 ± 10.0	33.3 ± 13.3	24.8 ± 8.5	<0.001
Duration of hospitalization (days)		8.4 ± 4.4	5.2 ± 2.1	4.4 ± 2.4	2.6 ± 1.0	<0.001
Menses resuming after recovery (days)		15.8 ± 18.1	13.1 ± 2.8	13.5 ± 2.3	13.1 ± 6.1	0.390
Amount of bleeding (ml)		26.6 ± 5.1	25.9 ± 9.9	27.8 ± 12.1	24.2 ± 14.8	0.471

The cost-effectiveness ratio (C/E) was 1,876.53 yuan for the aspiration group, 2,164.63 yuan for the lauromacrogol group, 4,383.56 yuan for the local injection group, and 7,850.81 yuan for the UAE group. The ICER of the lauromacrogol group to the aspiration group was 5,477.75 yuan, indicating that if the willing to pay of patients was higher than 5,477.75 yuan, the lauromacrogol group had a cost-effectiveness advantage in treating type 1 CSP, compared to aspiration group. On the contrary, aspiration group has a higher cost-effectiveness advantage. The ICER of the lauromacrogol group to the local injection group or the UAE group were both less than 0, indicating that local injection group and UAE group was not cost-effective in the treatment of type 1 CSP (Table 2).

**TABLE 2:** Cost-effectiveness analysis of four treatments for type 1 CSP.

	Total treatment cost (yuan)	Effect	C/E (yuan)	ICER (yuan)
Aspiration group	1726.41	0.92	1876.53	0
Lauromacrogol group	2,164.63	1	2,164.63	5,477.75
Local injection group	3,111.01	0.71	4,383.56	−3,260
UAE group	7,752.67	0.98	7,850.81	−447043

**FIGURE 3:**
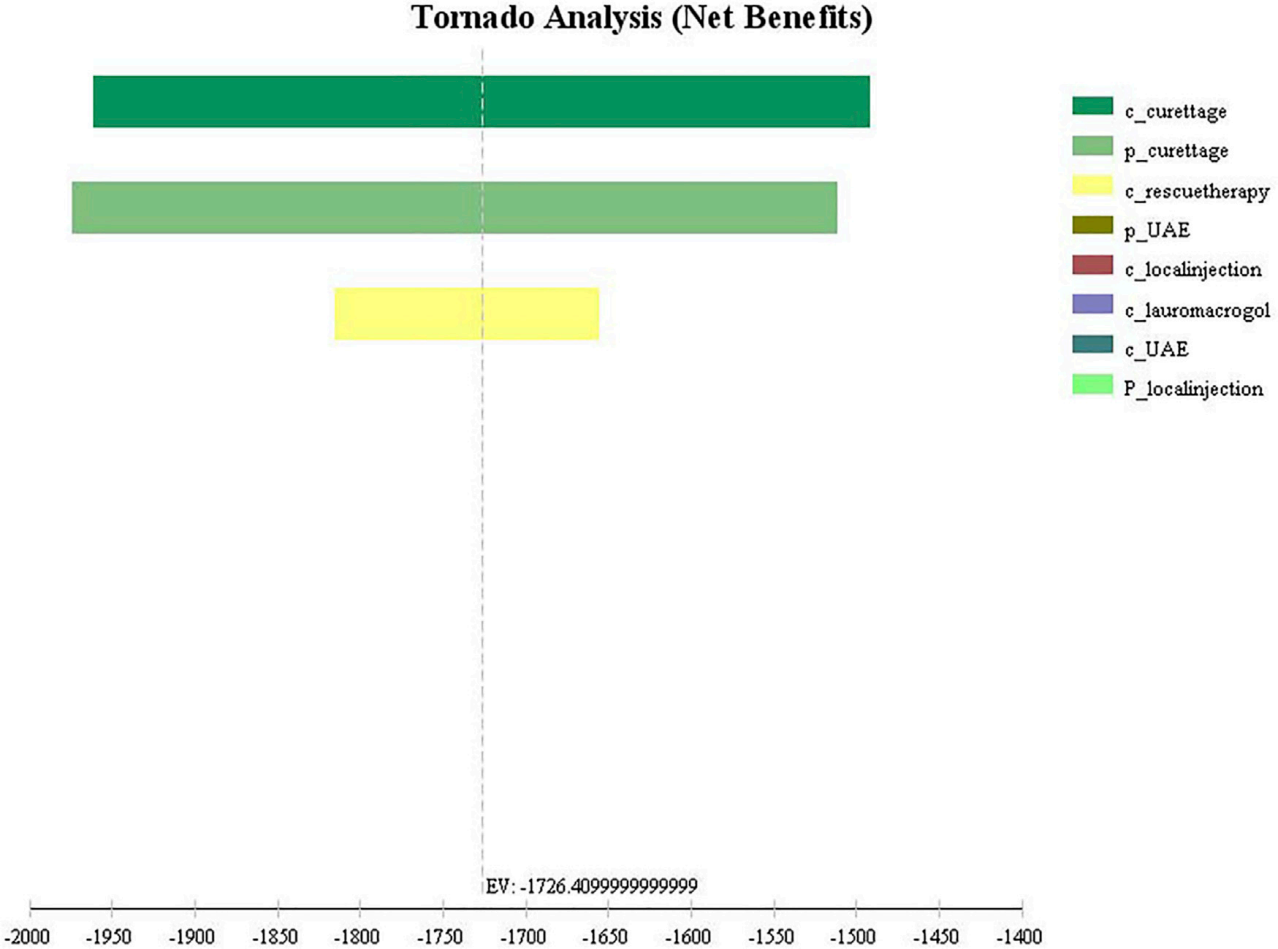
Tornado diagram of univariate sensitivity analysis for four treatments. c_curettage: cost of Aspiration group; p_curettage: success rate of Aspiration group; c_rescuetherapy: cost of rescue therapy; p_UAE: success rate of UAE group; c_local injection: cost of local injection group; c_lauromacrogol: cost of lauromacrogol group; c_UAE: cost of UAE group; p_local injection: success rate of local injection group.

**FIGURE 4:**
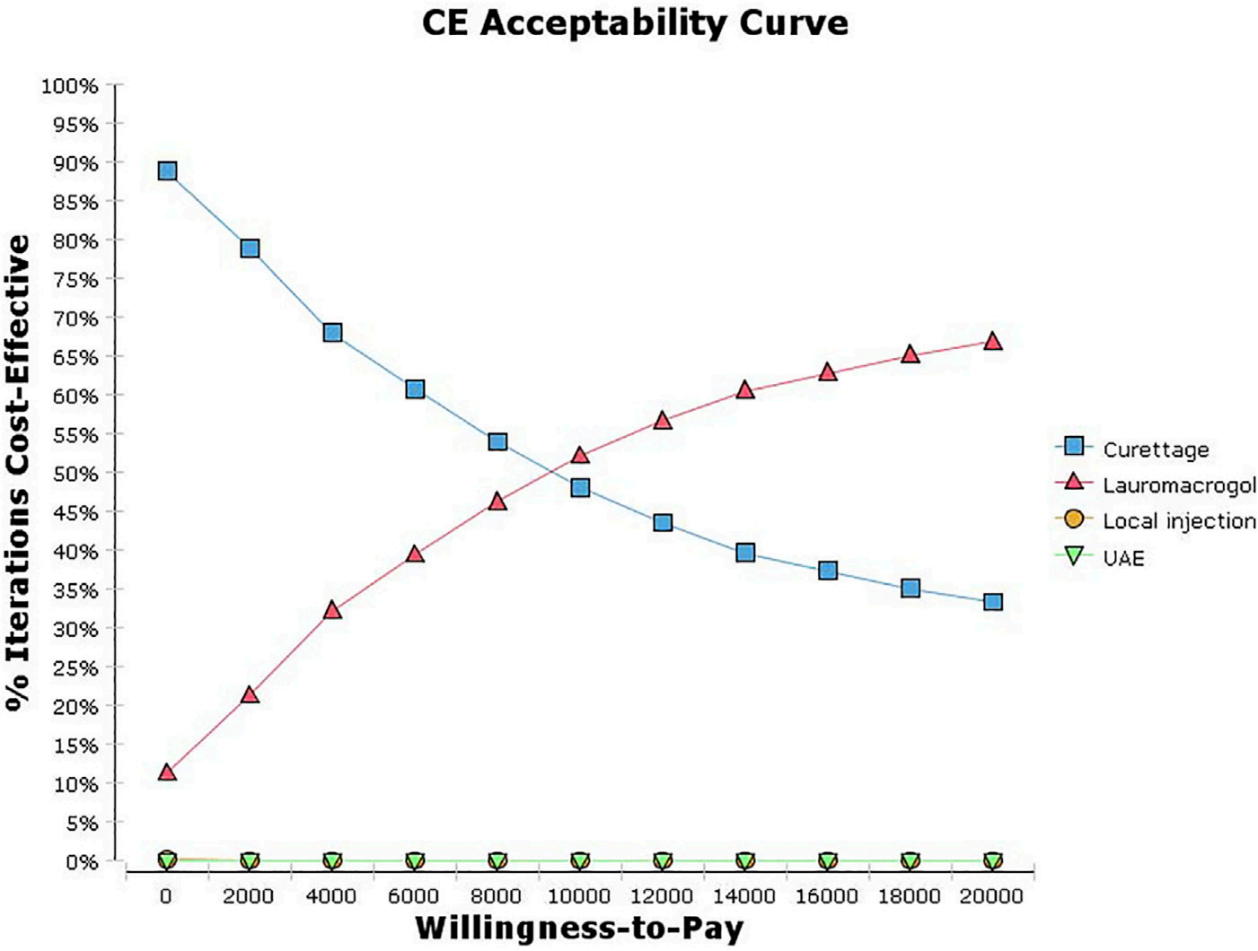
Cost-effectiveness acceptability curves of the four treatments.

The univariate sensitivity analysis can be seen from the results of the tornado diagram (Figure 3), the most influential factors are the treatment cost and success rate of the aspiration group, while other factors have little influence on the results. Moreover, the results are relatively robust, the outcome of the study has not been reversed within the variation range of each parameter (Supplementary Table S1). The probabilistic sensitivity analysis can be seen from the cost-effectiveness acceptable curve (Figure 4), with the increase in WTP, the cost-effectiveness acceptability of ultrasound-guided local injection of lauromacrogol combined with suction aspiration continues to rise, while the cost-effectiveness acceptability of ultrasound-guided suction aspiration continues to decline. However, ultrasound-guided local injection of MTX and uterine artery embolization combined with suction aspiration, are not cost effective.

## 4 Discussion

CSP is a special type of ectopic pregnancy in which placenta implantation and placenta previa may occur in the second and third trimester, leading to hysterectomy and even putting the life of the mother and fetus at risk. Therefore, pregnancy should be terminated as soon as one is diagnosed with CSP (Timor-Tritsch et al., 2019a). Several clinical parameters, including gestational age, β-HCG level, gestational sac diameter, and myometrium thickness in the c-section scar, have been proposed for the selection of CSP treatment options and to predict the risk factors for CSP treatment failure; however, the boundary values of these factors vary across studies (Liu et al., 2016; Chiang et al., 2017; Fang et al., 2017; Timor-Tritsch et al., 2021). Until now, there have been few studies on the treatment of CSP according to its classification. Our previous research shows that hysteroscopy-assisted laparoscopy is safe and effective for type 2 CSP (Qi et al., 2019), which has been confirmed by other similar studies (Wang et al., 2014; Shen et al., 2021). As the gestational sac is implanted on the scar and grows toward the uterine cavity, the risk of type 1 CSP is relatively lower, therefore, we evaluate the treatment of type 1 CSP based on the therapeutic effect and health economics.

Although dilatation and curettage alone—without adjuvant treatments—is associated with a high complication rate, and more than half of such patients require additional treatments (Birch Petersen et al., 2016), we should distinguish dilatation and curettage from suction aspiration. Ultrasound-guided suction aspiration has a good therapeutic effect and low complication rate; of the 191 women with CSP who received suction curettage, nine required a blood transfusion, one underwent hysterectomy, due to uncontrollable, intraoperative bleeding; and 7 out of 116 (6.0%) required additional treatment during follow up due to remnants (Jurkovic et al., 2016). In a study involving 36 CSP women who underwent suction curettage (Bağlı et al., 2021), the success rate was found to be 86% (31 cases); two cases required laparotomy, while three cases required additional treatment. It is worth noting that Foley balloon tamponade is an effective way to prevent and control bleeding during suction aspiration (Vo et al., 2019).

Lauromacrogol is widely used as a sclerosant, and the mechanism of its sclerosant effect has been described in our previous publication (Chai et al., 2018). To summarize, paravenous administration of lauromacrogol can cause venous fibrosis around the injection site, while direct intravascular injection of lauromacrogol can lead to local thrombogenesis (Eckmann et al., 2005; Parsi et al., 2008; Eckmann, 2009). Ultrasound-guided local injection of lauromacrogol combined with suction aspiration was first used for CSP in 2016 in our hospital, and achieved a good, therapeutic effect (Chai et al., 2018). Wu et al. (Wu et al., 2020) also verified that this treatment method had a high success rate (98.8%, 85/86) and a low complication rate (9.3%, 8/86). In this study, both aspiration group and lauromacrogol group had high success rate, short hospital stay and recovery time; the C/E for the aspiration group was lowest, the ICER of the lauromacrogol group to the aspiration group was 5,477.75 yuan, which means with the increase in WTP, the cost-effectiveness acceptability of ultrasound-guided local injection of lauromacrogol combined with suction aspiration continues to rise.

Ultrasound-guided local injection of MTX is the preferred medical treatment for CSP. In the review by Cheung (Cheung, 2015), 96 cases of CSP with local injection of MTX were studied, and the success rate reached 73.9% after a single injection of MTX, while it increased to 88.5% after an additional injection. In a systematic review by Birch Petersen (Birch Petersen et al., 2016), the success rate of local MTX injection was 64.9% with a complication rate of 4.1%, while the success rate of combined local and systemic MTX injection increased to 76.5% with a complication rate of 2.3%. However, Timor-Tritsch and Monteagudo’s review (Timor-Tritsch and Monteagudo, 2012) showed that local injection of methotrexate or KCl had one of the lowest complication rates among therapies (8/81 cases; 9.6%). They then conducted a retrospective study, reporting that 19 women with CSP who successfully received local and intramuscular MTX injection had an average recovery time of 88 days (24–177 days) (Timor-Tritsch et al., 2012). In this study, ultrasound-guided local injection of MTX had the lowest success rate of 71.0%, similar to the review by Cheung, and the longest hospital stay and recovery time. Meanwhile, the result of economics also indicated that ultrasound-guided local injection of MTX was not cost-effective in the treatment of type 1 CSP.

UAE can effectively reduce the risk of bleeding in the treatment of CSP (Shen et al., 2012; Qi et al., 2015; Birch Petersen et al., 2016). In our previous research, 792 cases involving UAE in the treatment of CSP had a high success rate of 93.06% and a very low hysterectomy rate of 1.64%. In a systematic review by Birch Petersen et al. ^[17]^, UAE in combination with dilatation and curettage and hysteroscopy (n = 85; 95.4% success, 1.2% complications), and UAE in combination with dilatation and curettage (n = 295; 93.6% success, 3.4% complications) were two treatments with a high success rate. However, the UAE treatment is expensive as it requires costly digital imaging equipment and specialized professionals. In addition, UAE can cause complications, such as lower limb arterial embolism and puncture point hematoma and may affect the patient’s future reproductive potential (Hois et al., 2008; Arthur et al., 2014). Our study shows that although the UAE group had a positive therapeutic outcome, its C/E was also the highest (7,850.81 yuan), and the ICER of the lauromacrogol group to the UAE group was less than 0, indicating that UAE combined with suction aspiration was not cost-effective in the treatment of type 1 CSP.

There are several limitations in our study. It retrospectively summarizes the treatment experience of our hospital in the past 10 years, so the practicality of the result need to be verified by multi-center, large-sample prospective studies. In addition, the effects of treatment on patients’ fertility and CSP recurrence risk need to be further studied.

In conclusion, for type 1 CSP, ultrasound-guided local injection of lauromacrogol combined with suction aspiration and ultrasound-guided suction aspiration, are both effective and economical, and the choice between the two can be based on the patient’s WTP.

## Data Availability

The raw data supporting the conclusion of this article will be made available by the authors, without undue reservation.
