# Supplementary material for: The Efficacy and Health Economics of Different Treatments for Type 1 Cesarean Scar Pregnancy
Source: Front Pharmacol. 2022 Jan 28;13:822319. doi: 10.3389/fphar.2022.822319 (PMC8831712; doi:10.3389/fphar.2022.822319)
Supplement: Supplementary file 1 [file Table1.DOCX]

Supplementary Material

**Supplementary Table 1. Univariate sensitivity analysis of the four treatments**

| Variation range of parameters | Group | Total treatment cost (yuan) | Effect | ICER (yuan) |
| --- | --- | --- | --- | --- |
| The cost of local injection group decreased to the 95%CI lower limit | Aspiration group | 1726.41 | 0.92 | 0 |
|  | Lauromacrogol group | 2164.63 | 1 | 5477.75 |
|  | Local injection group | 2802.22 | 0.71 | -2196.31 |
|  | UAE group | 7752.67 | 0.99 | -447043 |
| The cost of local injection group rose to the 95%CI upper limit | Aspiration group | 1726.41 | 0.92 | 0 |
|  | Lauromacrogol group | 2164.63 | 1 | 5477.75 |
|  | Local injection group | 3419.80 | 0.71 | -4323.70 |
|  | UAE group | 7752.67 | 0.99 | -447043 |
| The cost of UAE group decreased to the 95%CI lower limit | Aspiration group | 1726.41 | 0.92 | 0 |
|  | Lauromacrogol group | 2164.63 | 1 | 5477.75 |
|  | Local injection group | 3111.01 | 0.71 | -3260 |
|  | UAE group | 7561.26 | 0.99 | -431730 |
| The cost of UAE group rose to the 95%CI upper limit | Aspiration group | 1726.41 | 0.92 | 0 |
|  | Lauromacrogol group | 2164.63 | 1 | 5477.75 |
|  | Local injection group | 3111.01 | 0.71 | -3260 |
|  | UAE group | 7944.08 | 0.99 | -462356 |
| The cost of aspiration group decreased to the 95%CI lower limit | Aspiration group | 1491.70 | 0.92 | 0 |
|  | Lauromacrogol group | 2164.63 | 1 | 8411.63 |
|  | Local injection group | 3111.01 | 0.71 | -3260 |
|  | UAE group | 7752.67 | 0.99 | -447043 |
| The cost of aspiration group rose to the 95%CI upper limit | Aspiration group | 1961.11 | 0.92 | 0 |
|  | Lauromacrogol group | 2164.63 | 1 | 2544 |
|  | Local injection group | 3111.01 | 0.71 | -3260 |
|  | UAE group | 7752.67 | 0.99 | -447043 |
| The cost of lauromacrogol group decreased to the 95%CI lower limit | Aspiration group | 1726.41 | 0.92 | 0 |
|  | Lauromacrogol group | 2074.84 | 1 | 4355.38 |
|  | Local injection group | 3111.01 | 0.71 | -3569.30 |
|  | UAE group | 7752.67 | 0.99 | -454226 |
| The cost of lauromacrogol group rose to the 95%CI upper limit | Aspiration group | 1726.41 | 0.92 | 0 |
|  | Lauromacrogol group | 2254.42 | 1 | 6600.13 |
|  | Local injection group | 3111.01 | 0.71 | -2950.70 |
|  | UAE group | 7752.67 | 0.99 | -439860 |
| The success rate of local injection group decreased by 10% | Aspiration group | 1726.41 | 0.92 | 0 |
|  | Lauromacrogol group | 2164.63 | 1.00 | 5477.75 |
|  | Local injection group | 3302.34 | 0.64 | -3149.21 |
|  | UAE group | 7752.67 | 0.99 | -447043.20 |
| The success rate of local injection group rose by 10% | Aspiration group | 1726.41 | 0.92 | 0 |
|  | Lauromacrogol group | 2164.63 | 1.00 | 5477.75 |
|  | Local injection group | 2919.67 | 0.78 | -3442.50 |
|  | UAE group | 7752.67 | 0.99 | -447043.20 |
| The success rate of UAE group decreased by 10% | Aspiration group | 1726.41 | 0.92 | 0 |
|  | Lauromacrogol group | 2164.63 | 1.00 | 5477.75 |
|  | Local injection group | 3111.01 | 0.71 | -3260.00 |
|  | UAE group | 8018.90 | 0.89 | -52622.65 |
| The success rate of UAE group rose to 100% | Aspiration group | 1726.41 | 0.92 | 0 |
|  | Lauromacrogol group | 2164.63 | 1.00 | 5477.75 |
|  | Local injection group | 3111.01 | 0.71 | -3260.00 |
|  | UAE group | 7718.97 | 1.00 | 0 |
| The success rate of aspiration group decreased by 10% | Aspiration group | 1974.44 | 0.83 | 0 |
|  | Lauromacrogol group | 2164.63 | 1.00 | 1105.74 |
|  | Local injection group | 3111.01 | 0.71 | -3260.00 |
|  | UAE group | 7752.67 | 0.99 | -447043.20 |
| The success rate of aspiration group rose to 100% | Aspiration group | 1510.73 | 1.00 | 0 |
|  | Lauromacrogol group | 2164.63 | 1.00 | 0 |
|  | Local injection group | 3111.01 | 0.71 | -5512.50 |
|  | UAE group | 7752.67 | 0.99 | -499355.20 |
